# The Mutation Spectrum of Rare Variants in the Gene of Adenosine Triphosphate (ATP)-Binding Cassette Subfamily C Member 8 in Patients with a MODY Phenotype in Western Siberia

**DOI:** 10.3390/jpm13020172

**Published:** 2023-01-19

**Authors:** Dinara Ivanoshchuk, Elena Shakhtshneider, Svetlana Mikhailova, Alla Ovsyannikova, Oksana Rymar, Emil Valeeva, Pavel Orlov, Mikhail Voevoda

**Affiliations:** 1 Federal Research Center Institute of Cytology and Genetics, Siberian Branch of Russian Academy of Sciences, Prospekt Lavrentyeva 10, 630090 Novosibirsk, Russia; 2 Institute of Internal and Preventive Medicine—Branch of Institute of Cytology and Genetics, Siberian Branch of Russian Academy of Sciences, Bogatkova Str. 175/1, 630004 Novosibirsk, Russia

**Keywords:** maturity-onset diabetes of the young, MODY, diabetes mellitus, next-generation sequencing, *ABCC8*, SUR1, single-nucleotide variant

## Abstract

During differential diagnosis of diabetes mellitus, the greatest difficulties are encountered with young patients because various types of diabetes can manifest themselves in this age group (type 1, type 2, and monogenic types of diabetes mellitus, including maturity-onset diabetes of the young (MODY)). The MODY phenotype is associated with gene mutations leading to pancreatic-β-cell dysfunction. Using next-generation sequencing technology, targeted sequencing of coding regions and adjacent splicing sites of MODY-associated genes (*HNF4A*, *GCK*, *HNF1A*, *PDX1*, *HNF1B*, *NEUROD1*, *KLF11*, *CEL*, *PAX4*, *INS*, *BLK*, *KCNJ11*, *ABCC8*, and *APPL1*) was carried out in 285 probands. Previously reported missense variants c.970G>A (p.Val324Met) and c.1562G>A (p.Arg521Gln) in the *ABCC8* gene were found once each in different probands. Variant c.1562G>A (p.Arg521Gln) in *ABCC8* was detected in a compound heterozygous state with a pathogenic variant of the *HNF1A* gene in a diabetes patient and his mother. Novel frameshift mutation c.4609_4610insC (p.His1537ProfsTer22) in this gene was found in one patient. All these variants were detected in available family members of the patients and cosegregated with diabetes mellitus. Thus, next-generation sequencing of MODY-associated genes is an important step in the diagnosis of rare MODY subtypes.

## 1. Introduction

Maturity-onset diabetes of the young (MODY) is a rare monogenic type of diabetes mellitus with autosomal dominant inheritance and includes 14 subtypes, which are classified by causative genes: *HNF4A*, *GCK*, *HNF1A*, *NEUROD1*, *PDX1*, *HNF1B*, *KLF11*, *CEL*, *PAX4*, *INS*, *BLK*, *KCNJ11*, *ABCC8*, or *APPL1* [1]. The complexity of diagnosing MODY is due to the similarity of its clinical signs with type 1 diabetes mellitus (T1DM) and type 2 diabetes mellitus (T2DM) [2]. Patients with monogenic types of diabetes mellitus require a personalized approach to the selection of a proper treatment [3]. Verification of these types of diabetes is possible only through molecular genetic testing. Without this analysis, up to 80% of cases of monogenic diabetes can be misdiagnosed or may go undiagnosed [4]. Most MODY cases (70%) are due to mutations in the *GCK* or *HNF1A* gene, whereas pathogenic variants in other genes are rarer [5]. The gene of adenosine triphosphate (ATP)-binding cassette subfamily C member 8 (*ABCC8*) is reported to be associated with the MODY12 subtype, permanent or transient neonatal diabetes mellitus, and an opposite phenotype: hyperinsulinemic hypoglycemia [6,7,8]. This gene is located on the short arm of chromosome 11, consists of 39 exons, and encodes a protein of 1581 amino acid residues. The product of *ABCC8* is a sulfonylurea receptor (SUR1), a regulatory subunit of the ATP-sensitive K^+^ channel in membranes of pancreatic β-cells. This channel is composed of four inward-rectifier potassium ion pore-forming subunits (Kir6.2) and four SUR1 subunits combined into a hetero-octameric complex [9]. One of the main functions of this channel is the regulation of insulin secretion through changes in the membrane potential of the cell [10]. When the glucose level rises, the ATP/adenosine diphosphate (ADP) ratio increases in β-cells, thus leading to the closure of the K^+^ channel with the subsequent opening of a voltage-gated calcium channel. Insulin secretion goes up as a consequence [10]. An increase in ADP concentration influences SUR1 by forcing the channel to open and preventing an insulin release [11]. SUR1 is known to be a multidomain protein that includes transmembrane-domain 0 (TMD0, exons 1–4), loop 0 (L0, exons 5 and 6), transmembrane domain 1 (TMD1, exons 6–12), a part of nucleotide-binding domain 1 (NBD1, exons 13–15), and a sulfonylurea receptor motif (exons 2, 3, 5, and 7). Exons 17 to 39 code for P-loop-containing nucleoside triphosphate hydrolase, transmembrane domain 2 (TMD2), and nucleotide-binding domain 2 (NBD2) [12]. More than 400 mutations in the *ABCC8* gene have been described, most of which are located in coding parts of the gene (www.hgmd.org, accessed on 2 November 2022). Hyperinsulinism of various severity levels is usually induced by inactivating mutations in *ABCC8* [13]. Activating mutations in the *ABCC8* gene reduces the sensitivity of the channel to the inhibitory effect of ATP and enhances its sensitivity to ADP, thereby leading to the channel opening regardless of glucose levels. Such mutations can cause permanent or transient neonatal diabetes, MODY, or T2DM [14,15,16]. Most patients with *ABCC8* mutations have diabetes only; however, a greater decrease in the channel’s sensitivity to ATP gives rise to a more severe clinical phenotype, which may include neurological features, such as a developmental delay, seizures, epilepsy, mild dystonia, tonic posture, and muscle weakness [14,15].

The *ABCC8*-associated phenotype may depend on the type of mutation: variants activating the channel cause diabetes mellitus, whereas inactivating ones usually induce hyperinsulinism [13,14]. Few cases are described where the carriage of the same substitution in the same residue causes hyperglycemia or congenital hyperinsulinism in different patients [17,18]. There are also cases when congenital hyperinsulinism transforms into diabetes mellitus later in life [19,20,21]. An association of common variant rs757110 G of the *ABCC8* gene with the risk of T2DM has also been shown in the global population [22]. Clinical variability of symptoms and genetic heterogeneity of patients carrying mutations in the *ABCC8* gene complicates MODY12 diagnosis. Most cases of *ABCC8*-dependent diabetes are misdiagnosed as other types of diabetes mellitus, and insulin is mistakenly prescribed, which can result in poor control of carbohydrate metabolism [23]. Therefore, genetic testing is required for the identification of a causative gene in patients with a family history of diabetes.

In this study, the screening of rare genetic variants in *ABCC8* was performed using next-generation sequencing (NGS) technology in patients with hyperglycemia accompanied by the absence of antibodies against pancreas islet cells and glutamic acid decarboxylase and without ketoacidosis. The pathogenicity of the genetic variants was evaluated according to the standards of the American College of Medical Genetics (ACMG) and Genomics and the Association for Molecular Pathology [24], available databases, and literature data.

## 2. Materials and Methods

### 2.1. Study Subjects

The study protocol was approved by the local Ethics Committee of the Institute of Internal and Preventive Medicine (a branch of the Institute of Cytology and Genetics, the Siberian Branch of the Russian Academy of Sciences, Novosibirsk, Russia), protocol number 7 of 22 June 2008.

The total group of unrelated patients consisted of 285 persons (23.1 ± 11.7 years old [mean ± SD]; 37.9% males) examined at the Clinical Department of the Institute of Internal and Preventive Medicine from the year 2014 to 2022. Diabetes mellitus was diagnosed according to the criteria of the American Diabetes Association (Arlington County, VI, USA): HbA1C ≥ 6.5%, or fasting plasma glucose ≥ 126 mg/dL (7.0 mmol/L), or 2-h plasma glucose ≥ 200 mg/dL (11.1 mmol/L) during an oral glucose tolerance test (in the absence of unequivocal hyperglycemia; the result had to be confirmed by repeat testing), or a patient with classic symptoms of hyperglycemia or a hyperglycemic crisis with a random plasma glucose level ≥ 200 mg/dL (11.1 mmol/L) [25]; a debut of the disease in probands at the age of 35 years or earlier; a family history of diabetes mellitus; the absence of obesity; the absence of antibodies against pancreas islet cells and glutamic acid decarboxylase; intact secretory function of β-cells; normal or mildly reduced C-peptide levels; no need of insulin therapy; and the absence of ketoacidosis at the onset of the disease. The study population could include patients with MODY as well as T1DM patients with a negative antibody test result and an early onset of T2DM. Patients with clinical features of atypical diabetes mellitus (differing from those of T1DM and T2DM) and, in some cases, lacking a family history were included in this study [26]. Patients with tuberculosis or human immunodeficiency virus infection, as well as those who underwent antiviral therapy for hepatitis B or C, who abused psychoactive substances or alcohol within 2 years prior to the study, were excluded.

### 2.2. Sequencing of MODY-Associated Genes and Bioinformatic Analysis

After informed consent was obtained, venous blood (5 mL) was collected from all the studied patients. DNA was extracted from the venous blood using phenol–chloroform extraction [27]. The quantity and quality of the DNA were assessed on an Epoch microplate spectrophotometer (BioTek, Winooski, VT, USA). The first step of the preparation of a DNA library included DNA fragmentation using the KAPA HyperPlus Kit (Roche, Switzerland). SeqCap EZ Prime Choice Probes (Roche, Basel, Switzerland) were employed for NGS target enrichment. Targeted regions included coding regions and adjacent splicing sites of the following MODY-associated genes: *HNF4A*, *GCK*, *HNF1A*, *PDX1*, *HNF1B*, *NEUROD1*, *KLF11*, *CEL*, *PAX4*, *INS*, *BLK*, *KCNJ11*, *ABCC8*, and *APPL1*. The HyperCap Target Enrichment Kit (Roche, Switzerland) was used for the recovery of captured DNA regions. The quality of the analyzed DNA and of the prepared libraries was evaluated by means of a capillary electrophoresis system, Agilent 2100 Bioanalyzer (Agilent Technologies Inc., Santa Clara, CA, USA). The prepared DNA samples were sequenced on the Illumina MiSeq platform (Illumina, San Diego, CA, USA) at the multi-access center Proteome Analysis (Federal Research Center of Fundamental and Translational Medicine, Novosibirsk, Russia). Automated processing and annotation of the obtained NGS data were carried out on the NGS Wizard platform (genomenal.com, accessed on 19 May 2021).

Data on the clinical significance and pathogenicity prediction of the annotated single-nucleotide variants (SNVs), ClinVar and VarSome, and literature data were employed for the analysis. Allele frequencies were annotated using databases GnomAD v3.1.2 [28] and RUSeq, 2 November 2022 (http://ruseq.ru/). Variants described in ClinVar or VarSome or predicted in silico to be benign/likely benign, as well as variants with minor allele frequency higher than 0.01% according to gnomAD and RUSeq, were excluded from the analysis. The pathogenicity of new variants was assessed in accordance with the recommendations of the ACMG and Genomics and the Association for Molecular Pathology [24].

The present study is focused on the spectrum of rare variants in the *ABCC8* gene.

### 2.3. ABCC8 Confirmation Analysis

The detected substitutions c.970G>A (p.Val324Met), c.1562G>A (p.Arg521Gln), and c.4609_4610insC (p.His1537Profs*22) in the *ABCC8* gene (NM_000352.6) were verified by Sanger sequencing of the corresponding DNA fragments of the *ABCC8* gene in probands and their relatives available for the analysis. The oligonucleotide primers used are shown in Table 1. The design of the oligonucleotides was performed in the Primer-Blast software 19 May 2021 (https://www.ncbi.nlm.nih.gov/tools/primer-blast/). The sequencing reaction was performed on an ABI 3500 instrument (Thermo Fisher Scientific, Waltham, MA, USA) using the BigDye Terminator v3.1 Cycle Sequencing Kit (Thermo Fisher Scientific, USA) in accordance with the manufacturer’s protocol. The sequences were analyzed in Chromas, 2 June 2021 (http://technelysium.com.au/wp/) and Vector NTI^®^ Advance 11 (Thermo Fisher Scientific, USA) software; a fragment of the *ABCC8* gene (NG_008867.1) served as a reference sequence for alignment.

## 3. Results

The search for pathogenic variants was carried out in 14 MODY-associated genes. No such variants were identified in *PDX1, NEUROD1, KLF11, CEL, PAX4, INS, BLK, KCNJ11,* and *APPL1.* In total, 55 out of the 285 probands proved to be carriers of pathogenic or probably pathogenic (previously described in ref. [29,30] or new) variants in *GCK*, *HNF1A*, *HNF4A*, *HNF1B*, and *ABCC8* (Supplementary Materials; Table S1.). Among these 55, only 3 probands are carriers of rare variants in the *ABCC8* gene.

### 3.1. Variants in Genes GCK, HNF1A, HNF4A, and HNF1B

A total of 36 probands out of the 55 were found to be carriers of pathogenic and probably pathogenic variants (nonsense mutations, small deletions, missense mutations, or splice site mutations) in the *GCK* gene, and 13 probands are carriers of variants in the *HNF1A* gene.

Among the additionally examined patients, previously described *GCK* variants were identified: two patients (P398 and P412) turned out to be carriers of c.238G>A (p.Gly80Ser) in exon 2; c.556C>T (p.Arg186*) and c.562G>A (p.Ala188Thr) in exon 5 were found in probands P186 and P188, respectively; c.659G>A (p.Cys220Tyr) in exon 6 and c.683C>T (p.Thr228Met) in exon 7 was detected in probands P384 and P433, respectively (Supplementary Materials; Table S1). Novel dinucleotide deletion AC c.11_12del (p.Asp4Glufs*47) was revealed in one proband (P437) in exon 1 of the *GCK* gene (Supplementary Materials; Table S1).

In the *HNF1A* gene, novel variant c.335delA (p.Pro112Argfs*43) and previously described c.872dupC (p.Gly292Argfs*25) and c.872delC (p.Pro291Glnfs*51) were identified (Supplementary Materials; Table S1). Proband P73 is a carrier of the c.160C>T (p.Arg54*) variant in the *HNF1A* gene and c.1562G>A (p.Arg521Gln) in the *ABCC8* gene (Supplementary Materials; Table S1). Novel single-nucleotide deletion c.85delC (p.Asn30Thrfs*74) in the *HNF4A* gene was detected in a heterozygous state in one proband: P381. We described the patient’s medical history and clinical features in ref. [31].

Two unrelated participants with diabetes and negative for autoimmunity (P27 and P400) carry previously described variant c.1006C>A (p.His336Asp) in the *HNF1B* gene. Some variants of this gene are associated with the MODY5 subtype and congenital anomalies of the kidneys and urinary tract and, less often, of the pancreas or genitalia [32]. In P27′s family, variant p.His336Asp did not cosegregate with a pathological phenotype. Family members of P400, other than the healthy mother, were not available for genetic analysis, and we had no information about any kidney or other anomalies among them. There are no published data with clear evidence of p.His336Asp pathogenicity [33,34], and it has been classified as a variant of uncertain significance in the LOVD database or a variant with conflicting interpretations of pathogenicity in ClinVar.

### 3.2. Variants in ABCC8

Because the MODY12 subtype is extremely rare in most populations [35], it is of interest to analyze variants in the *ABCC8* gene in patients with diabetes mellitus.

Earlier, we published a detailed clinical case of proband P12 [36].

The *ABCC8* gene variants identified in this study in the 285 probands (including three rare variants detected in this study) are presented in Table 2. Some common variants of this gene are reported in the literature to be associated with T2DM, but these results are ethnospecific [37]. No potentially pathogenic variants were identified here in adjacent regions of splice sites of this gene. Variants c.354C>T (p.Val118=), c.1678G>A (p.Val560Met), and c.2274G>A (p.Ala758=) were not included in the analysis because they were identified as benign in ClinVar (Variation ID: 255930, 188919, and 1097104, respectively) and in VarSome. Three heterozygous variants c.970G>A (p.Val324Met), c.1562G>A (p.Arg521Gln), and c.4609_4610insC (p.His1537ProfsTer22) were selected for further analysis.

### 3.3. The Phenotype of Patients with MODY12

Diabetes mellitus was diagnosed at ages of up to 27 in probands and up to 50 among their family members. At the onset of the disease, fasting hyperglycemia was determined during a routine examination or during pregnancy. There were no islet cell cytoplasmic antibodies (ICA), insulin antibodies (IAA), antibodies to glutamate decarboxylase (GAD), tyrosine phosphatase (IA2), antibodies to the zinc transporter (ZnT8A), and there were no symptoms of ketoacidosis at the onset of the disease. The weight of all patients was within the age norm (body–mass index (BMI): 18.2–22.6). In all the families examined, there was a family history of pathology of carbohydrate metabolism.

**Figure 1:**
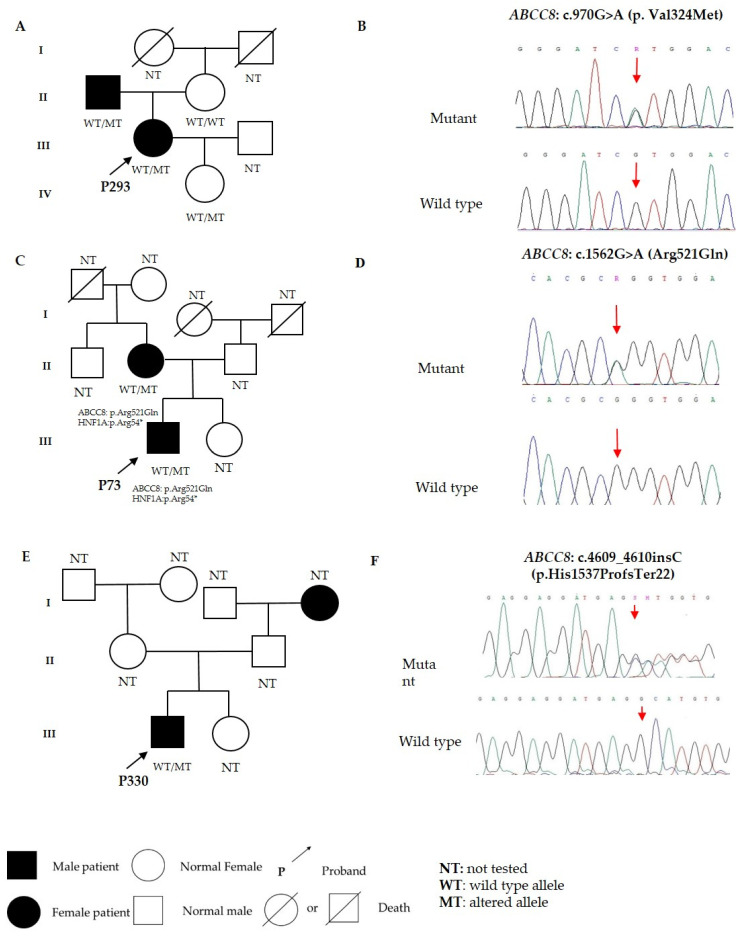
The screened families with identified variants. (**A**,**B**) c.970G>A (p.Val324Met), (**C**,**D**) c.1562G>A (p.Arg521Gln), and (**E**,**F**) c.4609_4610insC (p.His1537Profs*22) in the *ABCC8* gene.

When observed for 3 years (2019–2022), hyperglycemia ranged from asymptomatic to significant decompensation of carbohydrate metabolism in the probands. Macro- and microvascular complications were not detectable at the time of examination and observation. All three probands (P293, P73, and P330) were on insulin therapy. Available family members of the three probands were examined for the presence of corresponding variants in the *ABCC8* gene (Figure 1).

Heterozygous missense mutation c.970G>A (p.Val324Met) in *ABCC8* was identified in proband P293, her affected father, and little daughter (Figure 1A,B) and was absent in the proband’s healthy mother. Close monitoring of carbohydrate metabolism parameters in proband P293′s daughter was recommended because of the high risk of diabetes mellitus. We did not find other pathogenic variants in other MODY-associated genes in the proband. This variant is classified as pathogenic in ClinVar (Variation ID: 1338342) and VarSome (ACMG: PS3, PP3, PP5, PM1, and PM2). The variant was absent in databases gnomAD and RUSeq (Table 2).

Heterozygous missense mutation c.1562G>A (p.Arg521Gln) in the *ABCC8* gene was identified in proband P73 and his affected mother (Figure 1C,D). Other family members were unavailable for the examination. The proband and his mother also proved to be carriers of pathogenic variant c.160C>T (p.Arg54*) in the *HNF1A* gene [29]. Arg521Gln in the *ABCC8* gene is classified as “conflicting interpretations of pathogenicity” in ClinVar (Variation ID: 157683) and as “Uncertain Significance” in VarSome (ACMG: PM1, PM2, PP5, and BP4). This variant is described in gnomAD v3.1.2 with minor allele frequency (MAF) = 0.0001117 and RUSeq (MAF) = 0.0004160, eastern Russia) (Table 2). In both databases, this variant is described only in a heterozygous state. In the proband and his mother, the variant cosegregated with the disease (criterion PP1).

In proband P330, the c.4609_4610insC (p.His1537Profs*22) variant of the *ABCC8* gene was identified (Figure 1E,F). It is not described in databases gnomAD v3.1.2 and RUSeq (criterion PM2) or in the literature. The patient’s relatives were not available for the analysis. The c.4609_4610insC variant (p.His1537Profs*22) is a single-nucleotide deletion of cytosine that results in a frameshift and probably a premature stop codon. Loss-of-function mutations in the *ABCC8* gene have been repeatedly described and are pathologically significant (criterion PVS1). In silico analysis showed that the variant is damaging (criterion PP3). Thus, according to the set of criteria (PVS1, PM2, and PP3), p.His1537Profs*22 was assumed to be pathogenic.

## 4. Discussion

MODY was suspected in the probands owing to the age of onset of diabetes mellitus before 45 years, the presence of relevant family history, the absence of ketoacidosis at the onset of the disease, the absence of relevant antibodies, and the absence of symptoms of insulin resistance. We did not find any MODY12-specific symptoms common among the three probands and their relatives other than the usual clinical signs of MODY.

The c.970G>A (p.Val324Met) substitution is located in the transmembrane domain TMD1 of SUR1. There is a report of heterozygous carriage of this mutation in a female patient (age at diagnosis: 2 days) with transient neonatal diabetes mellitus without relapse at the time of examination (22 weeks) [47]. The substitution was inherited on the maternal side, but the proband’s mother had no signs of diabetes. A male patient with neonatal diabetes and a severe developmental delay at 74 days of life has been described who carries substitutions c.970G>A (p.Val324Met) and Arg1394Leu in the *ABCC8* gene; at the age of 6, he still had diabetes (C-peptide level 0.07, HbA1c 61 mmol/mol, and the absence of relevant antibodies), and his treatment was changed to sulfonylureas [38]. Carriage of a c.970G>A (p.Val324Met) and the Trp688Arg compound heterozygous variant in the *ABCC8* gene was associated with permanent neonatal diabetes mellitus in a 17-year-old Italian female (age of manifestation: 15 days) [39]. A successful treatment change from insulin to sulfonylureas was reported. It is likely that the patient’s deceased mother was a carrier of the c.970G>A (p.Val324Met) variant because the 74-year-old grandfather of the proband is a carrier of this variant and has diabetes mellitus (2 h plasma glucose: 14 mmol/L). The paternal grandmother of the proband is a carrier of the Trp688Arg variant and features impaired glucose tolerance (2 h plasma glucose: 8.4 mmol/L) [39]. A heterozygous male carrier of c.970G>A (p.Val324Met) with transient neonatal diabetes mellitus (absence of relevant antibodies, glucose at 24 mmol/L, and presence of ketoacidosis) and a developmental speech delay has been described [40]. The diabetes relapsed at age 9, and treatment with insulin was prescribed. After identification of the mutation, the treatment was switched successfully to glibenclamide [40]. Functional studies on a cell line have shown that the c.970G>A (p.Val324Met) variant causes a severe activating gating defect and reduces SUR1 expression on the cell surface, followed by attenuation of its functional effect on β-cells [48].

Variants responsible for the development of late-onset autosomal dominant diabetes in genes of ATP-sensitive K^+^ channels have rarely been described [42]. Heterozygous variant c.1562G>A (p.Arg521Gln) in the *ABCC8* gene has been found in a man with nonimmune diabetes mellitus and a family history of diabetes; the age of manifestation is 34 [41]. The same variant has been identified by laboratory tests in another person with diabetes mellitus [42]. The same as p.Val324Met, p.Arg521Gln is located in transmembrane domain 1 of the SUR1 protein; this domain is involved in ATP binding. In proband P73 and his mother (Figure 1C), this variant was found to be combined with a substitution in the *HNF1A* gene. In terms of the clinical phenotype, carriers of pathogenic *ABCC8* gene variants are similar to patients with *HNF1A* and *HNF4A* MODY [7]; therefore, it was not possible to identify a contribution of a specific mutation to the clinical signs.

Heterozygous variant c.4609_4610insC (p.His1537Profs*22) was found in proband P330. During the survey, a family history of disorders of carbohydrate metabolism was revealed (Figure 1E), but his relatives were not available for the analysis. The detected variant is located in NBD2, which is responsible for the binding of Mg-nucleotides and, as a result, channel opening and membrane hyperpolarization, which leads to the prevention of insulin secretion. Known mutations in *ABCC8* that cause diabetes mellitus either increase the activation of the Mg-nucleotide-mediated channel or alter the intrinsic gating [49]. Functional studies on this variant have not yet been conducted, and there are no data on this variant in the literature and databases.

Thus, in patients with a MODY phenotype in the Russian population, 18 previously described and one novel [c.4609_4610insC (p.His1537ProfsTer22)] variant as revealed in the *ABCC8* gene. Among them, we identified four potentially causative variants [c.970G>A (p.Val324Met), c.1562G>A (p.Arg521Gln), c.4369G>C (p.Ala1457Thr), and c.4609_4610insC (p.His1537Profs*r22)], which cosegregated with diabetes mellitus in the available family members of the patients.

### Limitations

This study has some limitations due to the unavailability of information about some family members.

## 5. Conclusions

Our results suggest that variants c.970G>A (p.Val324Met), c.1562G>A (p.Arg521Gln), and c.4609_4610insC (p.His1537ProfsTer22) in *ABCC8* could be the cause of MODY-*ABCC8* in the Russian population. We did not detect any specific clinical features of MODY among patients carrying pathogenic variants of *ABCC8*, thereby confirming the need for genetic testing of patients with a MODY phenotype using NGS for correct diagnosis and treatment as well as for counseling the patients’ relatives.

## Figures and Tables

**Table 1:** Sequencing primers used with the identified variants.

SNV	Forward Primer 5′-3′	Revers Primer 5′-3′	Product Length
c.970G>A (p.Val324Met)	GCCCAGCCGTGAATTAGCC	CCTCTGGCATTTCTGTTGACCA	429
c.1562G>A (p.Arg521Gln)	CTTTGAGTAGGCCACTTCACCT	CAGAGCCAGTTTGAGGCTCC	501
c.4609_4610insC (p.His1537Profs*22)	CCTGTCCCAAGGCCTTATATGT	GTATGGGCAGGGTCCGAATG	502

**Table 2:** The genetic variants in *ABCC8* exons identified in our patients, their minor allele frequencies in our group of patients and according to the gnomAD and RUSeq databases, and associated phenotypes according to the literature data.

dbSNP ID	Substitution (NM_000352.6)	Nucleotide Changes (NM_000352.6)	Minor Allele Frequency (Our Study)	Minor Allele Frequency (gnomADv3.1.2)	Minor Allele Frequency (RUSeq)	Associated Phenotype [Reference *]
rs1048099	p.Pro69=	c.207T>C	0.468	0.459	0.478	-
rs8192695	p.Ala110=	c.330C>T	0.046	0.065	0.043	-
rs137873871	p.Val118=	c.354C>T	0.008	0.004	0.009	-
rs2301703	-	c.579 + 14C>T	0.390	0.470	0.384	-
rs1328072266	p. Val324Met	c.970G>A	0.002	-	-	ND/TND [38,39,40]
rs368114790	p.Arg521Gln	c.1562G>A	0.002	0.000	0.000	DM [41,42]
rs2074308	-	c.1672-74G>A	0.159	0.121	0.153	T2DM [43]
rs4148619	p.Val560Met	c.1678G>A	0.002	0.000	0.000	-
rs1799857	p.His562=	c.1686C>T	0.390	0.441	0.408	-
rs1799858	p.Lys649=	c.1947G>A	0.131	0.166	0.142	T2DM [43]
rs1799854	-	c.2117-3C>T	0.523	0.372	0.480	T2DM [44]
rs761258571	p.Ala758=	c.2274G>A	0.002	0.000	-	-
rs1801261	p.Thr759=	c.2277C>T	0.002	0.028	-	T2DM [45]
rs1805036	p.Leu829=	c.2485C>T	0.079	0.140	0.099	-
rs1799859	p.Arg1273=	c.3819G>A	0.295	0.387	0.273	T2DM [46]
rs757110	p.Ala1369Ser	c.4105G>T	0.605	0.712	0.622	T2DM [22]
rs72559717	p.Ala1457Thr	c.4369G>A	0.002	0.000	-	MODY [36]
New	p.His1537Profs*22	c.4609_4610insC	0.002	-	-	-
rs8192690	p.Val1572Ile	c.4714G>A	0.055	0.051	0.069	-

DM: diabetes mellitus, ND: neonatal diabetes, T2DM: type 2 diabetes mellitus, TND: transient neonatal diabetes mellitus; * an association is reported in the literature.

## Data Availability

The data presented in this study are available on request from the corresponding author.

## Supplementary Materials

The following supporting information can be downloaded at: https://www.mdpi.com/article/10.3390/jpm13020172/s1, Table S1: The genetic variants identified in Western Siberian patients with a phenotype of maturity-onset diabetes of the young (MODY) and new genetic variants.
