# Supplementary material for: The Mutation Spectrum of Rare Variants in the Gene of Adenosine Triphosphate (ATP)-Binding Cassette Subfamily C Member 8 in Patients with a MODY Phenotype in Western Siberia
Source: J Pers Med. 2023 Jan 19;13(2):172. doi: 10.3390/jpm13020172 (PMC9967647; doi:10.3390/jpm13020172)
Supplement: Supplementary file 1 [file jpm-13-00172-s001.zip › jpm-2120690-supplementary.pdf]

Table S1. The genetic variants identified in Western Siberia patients with a phenotype of maturity onset diabetes of the young (MODY) and new variants.

Patient ID, gender	Gene	Variant status	Nucleotide changes*	Amino acid changes	Location	Genotype	Minor allele frequency (gnomAD)	dbSNP ID	ClinVar variation ID	HGMD	Pathogenicity according to ACMG [24], evidence	LOVD Database ID	Segregation with phenotype in family
**P437, Female**	***GCK***	**Novel**	**c.11_12del**	**p.Asp4Glufs*47**	**Exon 1**	**Heterozygous**	**NA**	**NA**	**NA**	**NA**	**Pathogenic, PM2, PVS1, PP3, PP1**	**NA**	Yes
P6, Female	*GCK*	Known	c.106C>T	p.Arg36Trp	Exon 2	Heterozygous	0.000014	rs762263694	431973	CM940823	PR	GCK_000007	Yes
P50, Male						Heterozygous							Yes
P59, Female	*GCK*	Known	c.110T>C	p.Met37Thr	Exon 2	Heterozygous	NA	NA	NA	NA	PR	GCK_000100	Yes
P17, Female	*GCK*	Known	c.130G>A	p.Gly44Ser	Exon 2	Heterozygous	NA	rs267601516	76898	CM013265	PR	GCK_000029	Yes
P403, Male	*GCK*	Known	.c.208G>A	p.Glu70Lys	Exon 2	Heterozygous	NA	NA	NA	CM930294	PR	NA	Yes
P74, Female	*GCK*	Known	c.238G>A	p.Gly80Ser	Exon 3	Heterozygous	NA	rs1554335761	449415	CM970630	PR	NA	Yes
P412, Female						Heterozygous							Yes
P398, Female						Heterozygous							Yes
**P51, Male**	***GCK***	**Novel**	**c.424A>T**	**p.Lys142***	**Exon 4**	**Heterozygous**	**NA**	**NA**	**NA**	**NA**	**Pathogenic, PVS1, PM2, PM1, PP1**	**NA**	**Yes**
**P4, Male**	***GCK***	**Novel**	**c.436C>G**	**p. Leu146Val**	**Exon 4**	**Heterozygous**	**NA**	**NA**	**NA**	**NA**	**Pathogenic, PS1, PS3, PM2, PP1**	**NA**	**Yes**
P10, Male	*GCK*	Known	c.449T>A	p. Phe150Tyr	Exon 4	Heterozygous	NA	rs193922297	129142	CM097114	PR	NA	Yes

**P86, Female**	***GCK***	**Novel**	**c.517_520del**	**p.Ala173Glnfs*30**	**Exon 5**	**Heterozygous**	**NA**	**NA**	**NA**	**NA**	**Pathogenic, PVS1, PS1, PM2, PM4, PP1, PP3**	**NA**	**Yes**
P90, Male	*GCK*	Known	c.517 G>A	p.Ala173Thr	Exon 5	Heterozygous	NA	NA	NA	NA	Pathogenic, PS1, PM2, PM5, PP1, PP3	GCK_000217	Yes
**P80, Female**	***GCK***	**Novel**	**c.542T>A**	**p.Val181Asp**	**Exon 5**	**Heterozygous**	**NA**	**NA**	**NA**	**NA**	**Pathogenic, PS1, PM2, PM5, PP1, PP3**	**NA**	**Yes**
**P46, Female**						**Heterozygous**							**Yes**
P428, Female	***GCK***	Known	c.556C>T	p.Arg186*	Exon 5	Heterozygous	NA	rs104894006	16133	CM920304	PR	GCK_000060	**Yes**
P453, Male	***GCK***	Known	c.562G>A	p.Ala188Thr	Exon 5	Heterozygous	0.000004	rs751279776	804849	CM930299	PR	GCK_000110	Yes
P14, Female	*GCK*	Known	c.580-1G>A	-	Intron 5	Heterozygous	NA	rs1554335421	449414	CS052048	PR	NA	Yes
P384, Female	*GCK*	Known	c.659G>A	p.Cys220Tyr	Exon 6	Heterozygous	NA	rs193922316	36240	CM121473	PR	GCK_000161	Yes
P30, Female	*GCK*	Known	c.660C>A	p.Cys220*	Exon 6	Heterozygous	NA	NA	NA	CM020443	PR	NA	Yes
P433, Female	***GCK***	Known	c.683C>T	p.Thr228Met	Exon 7	Heterozygous	0.000004	rs80356655	16134	CM920305	PR	GCK_000236	Yes
P68, Female	*GCK*	Known	c.700 T>C	p.Tyr234His	Exon 7	Heterozygous	NA	NA	NA	CM096864	PR	NA	Yes
P40, Female	*GCK*	Known	c.752T>C	p. Met251Thr	Exon 7	Heterozygous	NA	rs193922326	36251	CM096876	PR	NA	Yes
P67, Male	*GCK*	Known	c.755 G>A	p.Cys252Tyr	Exon 7	Heterozygous	NA	NA	NA	CM021266	PR	NA	NA
P83, Male	*GCK*	Known	c.771 G>A	p.Trp257*	Exon7	Heterozygous	NA	NA	NA	NA	Reported in [30]	NA	NA
P3, Male	*GCK*	Known	c.772G>T	p.Gly258Cys	Exon 7	Heterozygous	NA	rs1583596378	804857	CM032578	PR	NA	NA

P77, Female													Yes
P374, Female	***GCK***												Yes
P377, Female	*GCK*												Yes
**P88, Female**	***GCK***	**Novel**	**c.782 G>C**	**p.Gly261Ala**	**Exon 7**	**Heterozygous**	**NA**	**NA**	**NA**	**NA**	**Pathogenic, PS1, PM2, PM5, PP3**	**NA**	**NA**
**P54, Male**	***GCK***	**Novel**	**c.864 -1G>T**	**-**	**Intron 7**	**Heterozygous**	**NA**	**NA**	**NA**	**NA**	**Pathogenic, PVS1, PM2, PP1, PP3**	**NA**	**Yes**
**P57, Male**	***GCK***	**Novel**	**c.1113C>A**	**p.Cys371***	**Exon 9**	**Heterozygous**	**NA**	**NA**	**NA**	**NA**	**Pathogenic, PVS1, PS1, PM1, PM2, PM5, PP1**	**NA**	**Yes**
P70, Female	*GCK*	Known	c.1120G>A	p.Val374Met	Exon 9	Heterozygous	NA	rs1415041911	447380	CM096927	PR	NA	Yes
P61, Female	*GCK*	Known	c.1148C>A	p.Ser383*	Exon 9	Heterozygous	0.0000042	rs777870079	NA	CM032579	PR	NA	Yes
**P87, Male**	***GCK***	**Novel**	**c.1327 G>A**	**p.Glu443Lys**	**Exon 10**	**Heterozygous**	**NA**	**NA**	**NA**	**NA**	**Likely pathogenic, PM1, PM2, PP2, PP3**	**NA**	**NA**
**P19, Female**	***HNF 1A***	**Novel**	**c.18C>G**	**p.Ser6Arg**	**Exon 1**	**Heterozygous**	**NA**	**NA**	**NA**	**NA**	**Likely pathogenic, PS4, PM2, PP1, PP2, PP3**	**NA**	**Yes**
P11, Female	*HNF 1A*	Known	c.185A>G	p.Asn62Ser	Exon 1	Heterozygous	0.00012	rs377129682	447485	CM064300	PR	HNF1A_000235	NA
**P376, Female**	***HNF 1A***	**Novel**	**c.335delA**	**p.Pro112Argfs *43**	**Exon 2**	**Heterozygous**	**NA**	**NA**	**NA**	**NA**	**Pathogenic, PM2, PVS1, PP3, PP1**	**NA**	**Yes**

**P34, Female**	***HNF1A***	**Novel**	**c.526 +1 G>T**	**-**	**Intron 2**	**Heterozygous**	**NA**	**NA**	**NA**	**NA**	**Pathogenic, PVS1, PM2, PP1, PP2, PP3**	**NA**	**Yes**
P65, Female	*HNF1A*	Known	c.608G>A	p.Arg203His	Exon 3	Heterozygous	0.000008	rs587780357	129235	CM993816	PR	HNF1A_000137	Yes
**P91, Female**	***HNF1A***	**Novel**	**c.713G>A**	**p.Arg238Lys**	**Exon 3**	**Heterozygous**	**NA**	**NA**	**NA**	**NA**	**Likely pathogenic, PS1, PM2, PP2, PP3**	**NA**	NA
**P78, Male**	***HNF1A***	**Novel**	**c.713 +2 T>A**	**-**	**Intron 3**	**Heterozygous**	**NA**	**NA**	**NA**	**NA**	**Pathogenic, PVS1, PM2, PP1, PP2, PP3**	**NA**	**Yes**
P82, Female	*HNF1A*	Known	c.779C>T	p.Thr260Met	Exon 4	Heterozygous	0.0000040	rs886039544	265436	CM971457	PR	HNF1A_000148	NA
P408, Female	*HNF1A*	Known	c.872dupC	p.Gly292Argfs*25	Exon 4	Heterozygous	NA	rs587776825	14927	CI962354	PR	HNF1A_000160	Yes
P297, Female	*HNF1A*	Known	c.872delC	p.Pro291Glnfs*51	Exon 4	Heterozygous	NA	rs587776825	805637	CD972459	PR	HNF1A_000161	Yes
P16, Female	*HNF1A*	Known	c.1522G > A	p.Glu508Lys	Exon 6	Heterozygous	0.00044	rs483353044	135665	CM082841	PR	HNF1A_000214	NA
P81, Male	*HNF1A*	Known	c.1768G>A	p.Val590Met	Exon 9	Heterozygous	0.0000042	rs1168108747	447484	NA	Uncertain significance, PM2, PP1, PP2, PP3,	NA	Yes
P73, Male	*HNF1A*	Known	c.160C>T	p.Arg54*	Exon 1	Heterozygous	0.0000042	rs766956862	805632	CM032035	PR	HNF1A_000220	Yes
	ABCC8	Known	c.1562G>A	p.Arg521Gln	Exon 10	Heterozygous	0.000095	rs368114790	157683	CM1212138	PR	ABCC8_000172	
P293, Female	*ABCC8*	Known	c.970G>A	p.Val324Met	Exon 6	Heterozygous	NA	rs1328072266	1338342	CM073986	PR	NA	Yes
**P330, Male**	***ABCC8***	**Novel**	**c.4609_4610insC**	**p.His1537Profs*r22**	**Exon 39**	**Heterozygous**	**NA**	**NA**	**NA**	**NA**	**Pathogenic, PM2, PVS1, PP3, PP1**	**NA**	**NA**

P12, Male	*ABCC8*	Known	c.4369G>C	p.Ala1457Thr	Exon 36	Heterozygous	NA	rs72559717	NA	CM011260	PR	NA	Yes
P27, Female	*HNF1B*	Known	c.1006C>G	p.His336Asp	Exon 4	Heterozygous	0.0002	rs138986885	595653	CM067046	PR	HNF1B_000125	No
P400, Female						Heterozygous							NA
**P381, Female**	***HNF4A***	**Novel**	**c.85delC**	**p.Asn30Thrfs*74**	**Exon 3**	**Heterozygous**	**NA**	**NA**	**NA**	**NA**	**Pathogenic, PM2, PVS1, PP3.**	**NA**	**NA**

* RefSeq reference transcript: *GCK* (NM_000162.5), *ABCC8* (NM_000352.3), *HNF1B* (NM_000458.2), and *HNF1A* (NM_000545.6), *HNF4A* (NM_175914.5). HGMD: Human Genome Mutation Database; LOVD database: Leiden Open Variation Database; NA: not available; PR: previously reported to be associated with a pathological phenotype (in the literature, in LOVD, ClinVar, or HGMD) and was not classified according to ACMG recommendations. Variants identified for the first time are boldfaced.
